# War exposure, daily stressors, and mental health 15 years on: implications of an ecological framework for addressing the mental health of conflict-affected populations

**DOI:** 10.1017/S2045796024000830

**Published:** 2024-12-11

**Authors:** K. E. Miller, A. Rasmussen

**Affiliations:** 1 Education and Counselling Psychology and Special Education, The University of British Columbia, Vancouver, BC, Canada; 2 Psychology Department, Fordham University, New York, NY, USA

**Keywords:** armed conflict, daily stressors, ecological, mental health, trauma

## Abstract

**Aims:**

Fifteen years ago, we published an article in *Social Science and Medicine* seeking to resolve the contentious debate between advocates of two very different frameworks for understanding and addressing the mental health needs of conflict-affected populations. The two approaches, which we labelled *trauma-focused* and *psychosocial*, reflect deeply held beliefs about the causes and nature of distress in war-affected communities. Drawing on the burgeoning literature on armed conflict and mental health, the reports of mental health and psychosocial support (MHPSS) staff in the field, and on research on the psychology and psychophysiology of stress, we proposed an integrative model that drew on the strengths of both frameworks and underscored their essential complementarity. Our model includes two primary pathways by which armed conflict impacts mental health: directly, through exposure to war-related violence and loss, and indirectly, through the harsh conditions of everyday life caused or exacerbated by armed conflict. The mediated model we proposed draws attention to the effects of stressors both past (prior exposure to war-related violence and loss) and present (ongoing conflict, daily stressors), at all levels of the social ecology; for that reason, we have termed it an ecological model for understanding the mental health needs of conflict-affected populations.

**Methods:**

In the ensuing 15 years, the model has been rigorously tested in diverse populations and has found robust support. In this paper, we first summarize the development and key tenets of the model and briefly review recent empirical support for it. We then discuss the implications of an ecological framework for interventions aimed at strengthening mental health in conflict-affected populations.

**Results:**

We present preliminary evidence suggesting there has been a gradual shift towards more ecological (i.e., multilevel, multimodal) programming in MHPSS interventions, along the lines suggested by our model as well as other conceptually related frameworks, particularly public health.

**Conclusions:**

We reflect on several gaps in the model, most notably the absence of adverse childhood experiences. We suggest the importance of examining early adversity as both a direct influence on mental health and as a potential moderator of the impact of potentially traumatic war-related experiences of violence and loss.

Fifteen years ago, we published an article in *Social Science and Medicine* seeking to resolve the contentious debate between advocates of two very different frameworks for understanding and addressing the mental health needs of conflict-affected populations (Miller and Rasmussen, 2010a). The two approaches, which we labelled *trauma-focused* and *psychosocial*, reflect deeply held beliefs about the causes and phenomenology of distress in war-affected communities. They also differ regarding the types of interventions deemed most appropriate for strengthening mental health in conflict and post-conflict settings. Drawing on the burgeoning literature on armed conflict and mental health, the reports of mental health and psychosocial support (MHPSS) staff in the field, and on research on the psychology and psychophysiology of stress, we proposed an integrative model that drew on the strengths of both frameworks and underscored their essential complementarity.

We had little expectation that our paper would be widely read, or that the model we proposed would have any significant impact on a debate which had gone on for years (Tol *et al.*, 2013; Ventevogel, 2018; Wessells and van Ommeren, 2008). To our surprise, however, the model seemed to resonate widely. Although there have been several thoughtful critiques (Neuner, 2010; Tay and Silove, 2017), the response has been overwhelmingly positive. The paper earned *Social Science and Medicine’s* ‘top-cited paper’ award in 2014 and continues to be widely cited a decade later. In 2017, we published a companion paper in which we adapted the model to address the mental health needs of forcibly displaced populations (Miller and Rasmussen, 2017).That paper has had a similarly positive reception.

We recognize, of course, that citation counts say little about real-world impact. More specifically, they tell us nothing about the extent to which the model we proposed has had any discernible influence on policy and practice with conflict-affected populations – a critical point to which we return below. Here, we note merely that the model seemed to offer a welcome bridge between two competing yet fundamentally complementary frameworks for understanding and addressing mental health in humanitarian and refugee resettlement contexts.

In the past 15 years, the model has been rigorously evaluated in diverse conflict-affected populations. As we discuss below, support for it has been robust. Our primary aim in this paper, however, is to go beyond examining the statistical validity of the model and consider its implications for MHPSS interventions and the extent to which it has impacted MHPSS programming. In the process, we also consider areas of conceptual overlap and complementarity with the public mental health framework that has increasingly come to guide the development of MHPSS interventions in humanitarian settings (de Jong, 2002; Jordans and Kohrt, 2020; Purgato *et al.*, 2017; Tol *et al.*, 2015). Finally, we reflect on what we have learned about the limitations of the model and how these might be addressed.

## Origins: war exposure, daily stressors, and the need for an integrative framework

Here, we briefly summarize the key tenets of the trauma-focused and psychosocial frameworks. We refer readers interested in a more detailed description, as well as a discussion of the unique sociocultural and historical contexts that gave rise to each approach, to our earlier papers; see also the excellent discussion by Ventevogel (2018).

The trauma-focused framework prioritizes the adverse effects of prior exposure to war-related violence and loss. It is fundamentally a clinical model, rooted in the theories and methods of North American and European psychiatry and clinical psychology, and is focused on the psychotherapeutic and psychopharmacological treatment of trauma-related psychopathology. As such, it generally prioritizes intrapersonal mediators of change, such as the processing of trauma-related memories and affect, improving emotional regulation, and the alteration of maladaptive cognitions. In more recent years, the model has expanded beyond its historical focus on post-traumatic stress disorder (PTSD) to include a broader range of psychopathology but has largely retained its core assumption that distress is primarily rooted in exposure to the violence and destruction of armed conflict. For that reason, it may be more accurately termed a *war exposure* model (Miller and Rasmussen, 2017); we will refer to it as such throughout this paper.

In our critique of the war exposure model, we noted that it fails to account for the psychological impact of the harsh conditions of everyday life in conflict-affected settings – conditions that are either caused or exacerbated by armed conflict. We termed these conditions *daily stressors*. They include experiences such as extreme poverty and food insecurity, overcrowded and unsafe shelter, social isolation resulting from the loss of social networks, the marginalization and stigmatization of vulnerable groups, and an increase in all forms of family violence. We argued that the exclusion of such variables in the war exposure model had led to an overestimation of both the statistical and clinical significance of direct exposure to armed conflict, an overly narrow focus on healing what was presumed to be conflict-related trauma, and a failure to address current environmental stressors contributing to elevated levels of distress.

The psychosocial model, in contrast, draws attention to precisely those ongoing environmental stressors missing from the war exposure model. Psychosocial interventions focus primarily on altering the stressful conditions of everyday life in conflict-affected settings, conditions that may also impede natural processes of recovery from the effects of war-related violence and loss (Bonanno, 2004; Somasundaram *et al.*, 2023). Recognizing the difficulty of altering settings, psychosocial advocates also focus on fostering resilience to help people cope effectively with the harsh circumstances of daily life (Betancourt *et al.*, 2013; Tol *et al.*, 2013). Given the focus of the model on interpersonal and material mediators of change, it may more accurately be termed *social-environmental* – a label that also avoids the confusion arising from the varying uses of the term *psychosocial* in different sectors of the humanitarian world and in the related field of global mental health (Barbui *et al.*, 2020; Miller *et al.*, 2021; Ventevogel, 2018). We will use the label social-environmental in the remainder of this article.

As we noted in our 2010 paper, advocates of the social-environmental model, while drawing important attention to the psychological impact of ongoing environmental stressors, have historically tended to underestimate the painful reality of conflict-related trauma and related disorders, along with the need for clinical interventions for highly distressed individuals. In the various critiques of the war exposure model and its uncritical assumption of the universality and centrality of PTSD among survivors of armed conflict (Bracken *et al.*, 1995; Summerfield, 1999), there was often insufficient recognition that war-related trauma and traumatic grief reflect real and painful experiences for a substantial number of people, and that the severity of their symptoms can be effectively ameliorated with a variety of psychological therapies (Bangpan *et al.*, 2017; Liddell *et al.*, 2014; Purgato *et al.*, 2018).

**Figure 1.**
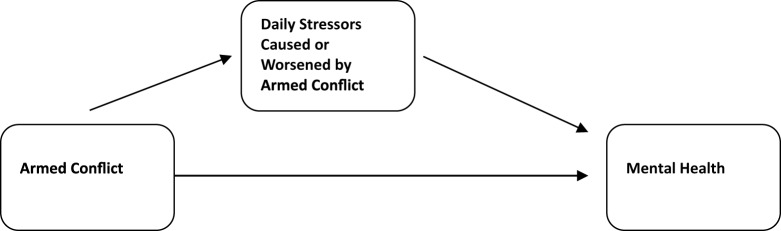
Ecological model of distress in conflict-affected populations.

*Note*: Adapted from Miller and Rasmussen (2010a)

The alternative model we proposed drew on a nascent but growing body of research examining the relative and interrelated contributions of war exposure and daily stressors to mental health status in conflict-affected populations. The model includes two pathways by which organized violence impacts mental health. Armed conflict exposes people directly to extreme violence and loss, thereby increasing the risk of PTSD and related disorders (e.g., prolonged grief); these are the direct effects of political violence on mental health at the core of the war exposure model. For that reason, we have also referred to the latter as a direct effects model. However, consistent with the social-environmental model, armed conflict also generates and exacerbates a host of chronic stressors rooted in the social ecology of everyday life. These *daily stressors* partially mediate (explain) the impact of armed conflict on mental health, and consistently account for equal or greater variance in mental health outcomes relative to direct exposure to the violence of war (Hou *et al.*, 2020; Miller and Rasmussen, 2010a, 2014, 2017; Roberts and Browne, 2011). The model we proposed was therefore a partially mediated model, which captures both the direct and indirect effects of conflict on mental health (see Fig. 1).

We continue to hear references to our model as a ‘daily stressors model’; in fact, we have occasionally referred to it this way ourselves (Rasmussen *et al.*, 2018). This is understandable, given the central role of daily stressors in the model, both as direct predictors of distress and as mediators of the effects of war exposure on mental health. However, the term is fundamentally inaccurate. Our model incudes multiple causal pathways, with daily stressors representing a constellation of risk factors that form a significant mediating pathway from armed conflict to mental health. To be clear, war exposure retains a direct effect on mental health (albeit a significantly weaker effect due to the partial mediation by daily stressors). The painful and enduring effects of direct exposure to war’s destructive power still constitute a significant pathway in the model – as one would expect for those exposed to the horrors of armed conflict.

The mediated model we proposed draws attention to the effects of stressors both past (prior exposure to war-related violence and loss) and present (ongoing conflict, daily stressors), at all levels of the social ecology (Bronfenbrenner, 1977). For this reason, in our adaptation of the model to the experience of internally displaced and refugee populations, we referred to it as an *ecological model* of distress (Miller and Rasmussen, 2017). Within an ecological framework, war exposure and social-environmental approaches are not in conflict; rather, they address the effects of temporally different stressors at different levels of the social ecology. This is precisely the sort of multilevel framework delineated by the Interagency Standing Committee (IASC) in its critically important document *Mental Health and Psychosocial Support in Emergency Settings* (Inter Agency Standing Committee, 2007) which sought to achieve a reconciliation between advocates of trauma-focused and psychosocial approaches three years before we first proposed our model. There is no question that the positive reception to the model we proposed has been due in no small part to the multilevel (‘pyramid’) approach already advocated by the IASC. In fact, the model we delineated can be seen as an empirically grounded, conceptual rearticulation of the IASC framework.

The model and the research underlying it suggest two important conclusions: (1) the stressful conditions of everyday life in conflict, post-conflict, and refugee settings are themselves highly distressing and even pathogenic – a finding consistent with research on the social determinants of mental health (Lund *et al.*, 2018); and (2) persistently stressful environments leave people more vulnerable to developing conflict-related PTSD and other psychiatric disorders (Somasundaram *et al.*, 2023) as well as culturally specific constructs of distress (Kohrt *et al.*, 2013). This may be due to the overwhelming of coping resources by perpetually elevated stress, which increases vulnerability to both acute and chronic psychological disorders (Kubiak, 2005; Sapolsky, 2004).

### Response to the proposed model

In critiquing the war exposure and social-environmental models and proposing an integrated framework that drew on the strengths of each, we felt some trepidation. Although the data supporting the ecological model that we presented were compelling, research on the relative contribution of war exposure and daily stressors to mental health was still somewhat sparse at the time; so too were studies examining the role of daily stressors in mediating the impact of armed conflict on mental health. We simply didn’t know how the model would hold up over time and with further evaluation. We also knew that the tension was palpable between advocates of the two approaches we sought to bridge, and that our discussion of the shortcomings of both models might not be well-received. Indeed, we had argued that both the war exposure and social-environmental frameworks were based on partial and selective readings of the research, and on research designs that failed to include critical sources of psychological distress in conflict, post-conflict, and refugee settings. Finally, the war exposure model had by that time enjoyed more than two decades as the dominant conceptual framework in research and practice with conflict-affected populations. In our model, the focus on war trauma and related psychiatric disorders was retained but significantly reduced. This challenged the historical dominance of the war exposure model and narrowed the role of trauma specialists in addressing mental health in conflict-affected populations.

To our relief, the model we had delineated seemed to resonate with both researchers and practitioners who had increasingly come to see the limitations of focusing narrowly on the effects prior war exposure, or conversely, of only addressing the effects of current environmental stressors. In a 2014 update to our original paper, we found robust support for the model in the literature (Miller and Rasmussen, 2014). By then, the model had been tested in a variety of settings and populations, using a diverse range of methodologies. Daily stressors consistently accounted for equal or greater variance in mental health outcomes relative to war exposure, and their inclusion resulted in a substantially greater amount of explained variance in psychological distress than models predicting mental health status solely based on prior war exposure.

In the ensuing years, the robustness of these findings has been further supported (Blackwell *et al.*, 2024; Bryant *et al.*, 2018; Hou *et al.*, 2020; Huțul *et al.*, 2023; Newnham *et al.*, 2015; Razjouyan *et al.*, 2022; Sim *et al.*, 2018; Somasundaram *et al.*, 2023), and they now include both longitudinal and experimental data (Bryant *et al.*, 2022, 2018; Jordans *et al.*, 2023). Mediation analyses have continued to find that the addition of daily stressors to the war exposure model results in a significant reduction in the strength of the direct effect of war exposure on mental health (Hou *et al.*, 2020; Newnham *et al.*, 2015; Ponnamperuma and Nicolson, 2018; Sim *et al.*, 2018); in fact, in some studies daily stressors have been found to fully mediate that effect (Hou *et al.*, 2020; Somasundaram *et al.*, 2023). For example, Tay, who wrote a commentary questioning the validity and clinical utility of distinguishing between war exposure and daily stressors (Tay and Silove, 2017), recently co-authored a study documenting the role of daily stressors in *fully mediating* the relationship of war exposure to psychological trauma among internally displaced, war-affected civilians in Sri Lanka (Somasundaram *et al.*, 2023).

It is by now clear that we simply cannot understand the impact of armed conflict on mental health without considering the stressful conditions of everyday life to which it gives rise. We turn now to a consideration of the implications of this ecological perspective for addressing mental health needs in conflict-affected populations.

## Implications of an ecological framework for MHPSS interventions

The primary aim in our 2010 paper was to broaden awareness of the multiple, interrelated sources of stress and distress impacting conflict-affected populations. The model we proposed and the research underlying it clearly suggest the importance of moving towards ecological or multilevel approaches to addressing mental health in humanitarian and refugee resettlement contexts. Specifically:

(1) The field of MHPSS should continue moving towards more integrated or holistic programming, in which the clinical treatment of psychological distress, regardless of its aetiology, is provided alongside a range of interventions that target the social determinants of distress in humanitarian settings (i.e., what we have referred to as daily stressors).

In practice, this could take various forms. Examples include:

- Ensuring that clinical services for distressed individuals are coupled with social services to help people navigate the difficult conditions of everyday life in humanitarian and resettlement contexts. This multimodal approach (Nickerson *et al.*, 2011) could take place within sufficiently resourced multiservice organizations, or by fostering greater coordination among service providers from different humanitarian sectors – a key recommendation of the IASC guidelines.
- Developing multilevel interventions that target both setting-level and intrapersonal mediators of distress. This might include, for example, the pairing of cash transfer or livelihoods programmes with supportive counselling, stress management, caregiver support, or transdiagnostic interventions aimed at reducing distress such as the WHO’s PM+, which recently showed promising, albeit uneven, effects across a diverse range of conflict-affected and refugee settings (Acarturk *et al.*, 2024; Bryant et al., 2022; de Graaff *et al.*, 2024).
- Developing stepped-care models of support, in which promotive, preventive, and treatment programmes are offered in a coordinated way that matches people with the appropriate level and type of support.

(2) Just as there has been a shift towards trauma-informed services in social-environmental programming (Im *et al.*, 2021), there is a parallel need for increased awareness of the impact of daily stressors among providers of trauma-focused and other clinical services.

In practice, this means carefully assessing ongoing environmental stressors that may be causing or exacerbating psychological distress, and that may undermine the effectiveness of clinical treatment if they are not simultaneously addressed. It also means assessing ongoing sources of traumatic stress that may not be readily apparent, such as intimate partner violence (IPV) and child abuse, both of which are known to increase in humanitarian crises (Clark *et al.*, 2010; Rubenstein *et al.*, 2020). In effect, this is simply an argument that all MHPSS providers should have a solid understanding of the dual burden of war exposure and daily stressors and their impact on mental health. By extension, it also implies that intervention strategies should be based on theories of change that reflect a comprehensive assessment of factors impacting mental health in the target population (Miller *et al.*, 2021).

(3) Alongside the call for greater attention to assessing and addressing daily stressors, there is a need for greater methodological rigour in evaluating interventions that target them for change. In his commentary on our 2010 paper, Neuner (2010) appropriately critiqued the weak empirical base for social-environmental (psychosocial) interventions, a concern echoed 10 years later by Haroz and colleagues (Haroz *et al.*, 2020). Unlike Neuner, however, who used this as an argument for prioritizing empirically supported trauma-focused interventions, we suggested that a more compelling response would be to strengthen both the conceptual underpinnings and evaluative rigour that could provide stronger evidence for interventions targeting daily stressors (Miller and Rasmussen, 2010b).

## A diversity of voices advocating for ecologically influenced MHPSS programming

In describing what we perceive as a gradual shift towards ecological programming within the field of MHPSS in conflict, post-conflict, and refugee settings, we hasten to add that we have not been alone in advocating for such a shift (Betancourt *et al.*, 2013; Goodkind *et al.*, 2020; Hobfoll *et al.*, 2011; Jordans and Tol, 2018; Miller and Rasco, 2004; Salo and Bray, 2016; Saltzman *et al.*, 2003; Tol *et al.*, 2013). While we hope that our model has contributed to the increasing adoption of an ecological framework for addressing mental health in conflict-affected populations, any progress in this regard reflects the diversity of voices that have been advocating for it, including the IASC guidelines (Inter Agency Standing Committee, 2007).

We also note the conceptual overlap between the ecological model we have advocated, and the public health framework that has played an increasingly important role in shaping the development and evaluation of MHPSS interventions (de Jong, 2002; Purgato *et al.*, 2017; Tol *et al.*, 2015). Both frameworks emphasize the importance of addressing the social determinants of mental health, and of targeting mediators of mental health outcomes through interventions based on clearly articulated theories of change. Both frameworks also conceptualize the clinical treatment of severe distress as just one element of a broader approach to strengthening mental health, a shift from the previously dominant view of clinical interventions as the cornerstone of addressing mental health in conflict-affected populations. However, we also note a key difference between the two frameworks. The ecological model we have proposed serves primarily as a *roadmap* for the development of MHPSS interventions by delineating the various factors influencing mental health both temporally (i.e., past and present) and at all levels of the social ecology. In contrast, a public health framework offers a useful typology of complementary intervention approaches – promotion, prevention, and treatment – for addressing putative mediators of mental health identified through an ecological analysis. Thus, for example, whereas an ecological assessment may point to the powerful impact of IPV on the mental health of conflict-affected women and children (Clark *et al.*, 2010; Rubenstein *et al.*, 2020), a public health framework would map neatly onto this analysis by drawing attention to various intervention strategies aimed at reducing (preventing) the occurrence of IPV while also treating its enduring psychological effects (Miller *et al.*, 2021). We concur with Purgato and her colleagues that there is an essential complementarity to the ecological and public health frameworks (Purgato *et al.*, 2017).

Finally, we also acknowledge the influence of other conceptual frameworks, including Conservation of Resources Theory (Hobfoll *et al.*, 2011) and the Adaptation and Development After Persecution and Trauma (ADAPT) model (Silove, 2013), which likewise emphasize the importance of current life circumstances (e.g., post-migration difficulties among refugees) as well as prior war exposure when addressing the mental health needs of war-affected populations.

Although a systematic review of the evidence of a shift towards ecologically influenced MHPSS programming is beyond the scope of this paper, here we briefly discuss examples which suggest that such a shift is in fact underway.

### An increase in multi-service/multisectoral programming and multilevel interventions

Despite repeated calls for greater intersectoral coordination and the development of multilevel interventions (Inter Agency Standing Committee, 2007; Jordans *et al.*, 2016; Miller *et al.*, 2021; Tol *et al.*, 2013), the field of MHPSS has been slow to adopt such recommendations (Ventevogel, 2018). Nonetheless, we see nascent signs of a shift, in humanitarian settings as well as in refugee resettlement contexts in high-income countries.

One could easily underestimate the shift towards greater multi-service and multilevel programming by focusing solely on published trials of MHPSS interventions. The research literature remains heavily focused on trials of clinical interventions that focus narrowly on intrapersonal mediators of change, and to a lesser extent on social-environmental interventions that target one ecological level (e.g., school-based preventive or treatment-focused interventions for children). However, as Tol and colleagues have noted, ‘… the most rigorously studied interventions are not those most commonly implemented in humanitarian settings …’ (Tol *et al.*, 2020). If we broaden our view beyond the academic literature to the programming of international and local non-governmental organizations, a more encouraging picture emerges. Increasingly, such organizations are adopting multi-service and stepped-care models of support, from community-wide promotion and prevention activities, nutritional assistance, literacy and educational programming, and livelihoods training, to psychotherapeutic interventions for distressed individuals and families. Examples include War Child’s ecological system of care (Jordans *et al.*, 2018); the work of Doctors Without Borders (Médicins Sans Frontierès), which includes mental healthcare, material support, and advocacy alongside its renowned medical assistance (Médicins Sans Frontierès, 2024); and the International Rescue Committee, which provides a range of programmes from education, economic support, health assistance, and parenting support (International Rescue Committee, 2024). Torture treatment centres and refugee mental health clinics are similarly adopting multi-service or multimodal models, in which clinical treatment is accompanied by a host of social and legal services aimed at helping clients navigate a range of daily or post-migration stressors (Hamid *et al.*, 2019; Nickerson *et al.*, 2011).

### Multilevel interventions

Multilevel interventions are those that target more than one level of the social ecology simultaneously. For example, the WHO intervention Early Adolescent Skills for Emotions provides therapeutic group sessions for distressed early adolescents together with caregiver sessions aimed at creating a home environment conducive to improved adolescent wellbeing (Bryant *et al.*, 2022b; Dawson *et al.*, 2019). In a similar vein, O’Callahan and colleagues developed a multilevel intervention for traumatized Congolese girls who had survived sexual assault. Their programme included culturally adapted CBT to ameliorate trauma symptoms in the girls, coupled with separate sessions for their caregivers to help them better support their children’s psychological recovery (O’Callaghan *et al.*, 2013).

A different approach to multilevel programming entails providing psychological support (e.g., individual or group counselling) while simultaneously addressing one or more social determinants of distress. Examples include combining livelihood support or cash transfers to alleviate extreme poverty, with individual or group support to help people manage stress and lower distress, strengthen parenting, or reduce household violence (Asghar *et al.*, 2017; Bossuroy *et al.*, 2022; Schinina *et al.*, 2016). Blattman and Annan offer an example of a multilevel intervention that focused on the social and economic reintegration of former youth combatants in post-conflict Liberia (Blattman and Annan, 2011). The intervention provided a combination of psychosocial counselling, agricultural training, and financial support. Although the effects were modest and variable across outcomes, the programme was noteworthy both for its multilevel approach to the challenge of post-conflict reintegration, and for the methodological rigor of its evaluation in a highly challenging context. Another promising example of a multilevel intervention is the ALIVE project, an ongoing initiative in Colombia, Nepal, and South Africa which aims to prevent adolescent depression through a combination of poverty reduction and strengthening self-regulation (Lund *et al.*, 2023). In describing the rationale for their dual focus on intrapersonal and environmental factors, Lund and colleagues aptly note that interventions aimed at strengthening mental health in settings of ongoing adversity are likely to have a limited impact if they focus solely on change-the-person strategies while ignoring the influence of powerful environmental stressors.

Another approach to ecological programming entails single-level interventions that target outcomes at different levels of the social ecology. Sociotherapy in post-genocide Rwanda, for example, is a group-based intervention that has been shown to improve individual mental health while also repairing social bonds weakened or destroyed by the genocide (Jansen *et al.*, 2015). Another example is the Nurturing Families intervention recently piloted in Jordan, which provides distressed families with modules aimed at improving adolescent and caregiver mental health while also strengthening family functioning. The pilot RCT showed promising effects on all caregiver-reported outcomes, with adolescent-reported outcomes yielding a more mixed picture (Brown *et al.*, 2024).

Although our focus has been on multilevel interventions that target mediators of change both within and beyond the individual, we also note the development of interventions that have shown positive effects on individual mental health while only targeting levels of the social ecology beyond the individual. For example, Goodkind and her colleagues (2020) demonstrated the positive impact of a multilevel intervention on the mental health of refugees from the Middle East, Afghanistan, and the Great Lakes region of Africa now resettled in the US. Their Refugee Wellbeing Project reduced distress among participants by fostering greater empowerment, improving access to essential resources, reducing social isolation, enhancing meaningful social roles, and improving relations with members of the host community. Notably, mental health outcomes improved without any psychotherapeutic component, a finding consistent with research on the powerful influence of daily stressors on mental health.

### Small steps towards change

We are cautious about overstating the extent to which the field of MHPSS has embraced ecological interventions in humanitarian and resettlement contexts. We agree with Ventevogel (2018), who observed that the various conceptual frameworks addressing both war exposure and current stressors have yet to demonstrate the same level of influence on practice as they have had on research (e.g., epidemiological studies, research on predictors of mental health status, etc.). On the other hand, we note optimistically that the increase in ecological programming has reached the point where a systematic review of multilevel interventions in low- and middle-income countries is now underway (Prina *et al.*, 2024). That review, which includes studies with populations affected by armed conflict as well as other forms of adversity, will examine interventions that combine psychological treatment with components that address social determinants of mental health, akin to what we have been calling daily stressors. We find it encouraging that a sufficiently large number of multilevel interventions have now been developed and evaluated to merit such a review, something that would have been unthinkable until recently.

### Increased rigor in the evaluation of social-environmental interventions

Evidence of increased evaluative rigor can be seen across a wide variety of social-environmental interventions, from low-intensity approaches such as child friendly spaces (Hermosilla *et al.*, 2019) and psychological first aid (Wang *et al.*, 2024), to more complex interventions aimed at reducing household violence (Asghar *et al.*, 2017), reintegrating former child soldiers (Blattman and Annan, 2011; Kerig and Wainryb, 2013), supporting the social and psychological adaptation of refugees resettled in high income countries (Goodkind *et al.*, 2020), or improving refugee children’s mental health by strengthening their caregivers’ wellbeing and parenting (Betancourt *et al.*, 2020; Jordans *et al.*, 2023; Puffer *et al.*, 2017). Increasingly, the same degree of methodological rigour that has traditionally been found in trials of clinical interventions is evident in the evaluation of social-environmental programming, with RCTs and mixed-methods designs rapidly becoming the norm. Unquestionably, concerns persist about the methodological rigour of evaluations of MHPSS interventions, both social-environmental and clinical (Haroz *et al.*, 2020; Miller *et al.*, 2023). Nonetheless, it is encouraging that we have come a long way from a reliance on impressionistic assessments and uncontrolled studies of interventions targeting daily stressors.

## Gaps in the model

We have heard from colleagues about a variety of conceptual and empirical gaps in the ecological model we proposed. We have also come to see certain limitations ourselves with the passing of time and the increased sophistication of the literature on armed conflict and mental health. Here, we briefly address three concerns.

### The absence of moderators

Our aims in the model we proposed were to broaden the then-dominant view beyond the focus on the effects of direct war exposure, and to offer an integrative framework for understanding the dual and interrelated impact of war exposure and daily stressors on mental health. Within that framework, there is certainly a need to consider how different variables moderate the impact of different risk factors (war exposure, daily stressors) on psychological outcomes. Moreover, although we have focused on the role of daily stressors in mediating the impact of war exposure on mental health, elsewhere, we have discussed how daily stressors, whether related to armed conflict or largely independent of it (e.g., chronic health conditions, child labour, child marriage, oppressive gender-based restrictions) may also *moderate* that relationship by increasing vulnerability to potentially traumatic experiences of war-related violence (Rasmussen *et al.*, 2018).

Although we have not included the wide range of variables found to moderate the impact of war exposure and daily stressors on mental health (Roberts and Browne, 2011), we hope that our delineation of these key sources of stress and distress has contributed to an interest in exploring the myriad features of conflict-affected populations and settings, and of MHPSS interventions, that might moderate the relationships among the variables included in our model. This might be research examining moderators of the direct effects of war exposure or daily stressors on mental health, or might include moderators of the mediating effect of daily stressors on the impact of war exposure on mental health – that is, moderated mediation. For example, gender may moderate the degree to which daily stressors mediate the effect of war exposure on mental health. In societies with particularly divergent gender roles, it seems reasonable to posit that men and women experience war exposure and daily stressors quite differently. Traditional narratives for men involving protection and the accumulation of wealth are often challenged in wartime, which may mean that men focus more on the precipitous loss of wealth and masculinity that so often accompanies exposure to war trauma; traditional narratives involving caretaking and maintaining a home may be more salient for women, which may result in more (relative) concern about the immediate challenges in post-war displacement settings. Moderated mediation has become a relatively common statistical approach and can fruitfully be applied to questions concerning the mitigation of the various sources of distress in humanitarian settings. We also note with encouragement the growing use of large individual participant databases (e.g., Karyotaki *et al.*, 2023), which afford the statistical power to shed light on the role of potentially key moderators of the relationships among predictors of distress, and of intervention outcomes.

### The bidirectional influence of war exposure and daily stressors

All the arrows indicating causality in our model are unidirectional. However, there is clearly considerable bidirectional influence between the impact of daily stressors and the enduring effects of war exposure. Just as high levels of daily stress can undermine people’s resilience and leave them more vulnerable to developing war-related PTSD (Somasundaram *et al.*, 2023), chronic trauma or traumatic grief can compromise people’s ability to cope effectively with everyday life stressors (Neuner, 2010; Stepakoff *et al.*, 2006). The unidirectional arrows in our model were meant to indicate the primary direction of causality over time; however, we recognize the bidirectional influence among variables in the model.

### The influence of adverse childhood experiences (ACEs) on adolescent and adult mental health

In our view, the omission of adverse childhood experiences (ACEs) is a critical gap not only in our model, but in the literature on war and mental health more broadly. While studies of conflict-affected younger children are increasingly assessing ACEs both within and outside of the home (Backhaus *et al.*, 2024; Catani *et al.*, 2008; Fernando *et al.*, 2010; Panter-Brick *et al.*, 2009; Wood *et al.*, 2020), most studies of conflict-affected adolescents and adults focus on the two sets variables in our model: war exposure and current stressors. Only a few studies have also asked about early experiences of adversity unrelated or indirectly related to armed conflict (Okello *et al.*, 2014; Olema *et al.*, 2014; Saltzman *et al.*, 2003). The findings of these studies suggest that early adversity in the home, particularly child maltreatment, is strongly related to adolescent and adult PTSD and depression independently of the effects of war exposure and current stressors. This shouldn’t come as a surprise; research on ACEs in populations unaffected by armed conflict has documented a powerful impact of early adversity on subsequent mental and physical health (Edwards *et al.*, 2003; Felitti *et al.*, 1998) and an increased vulnerability to the impact of potentially traumatic events (Cabrera *et al.*, 2007).

**Figure 2.**
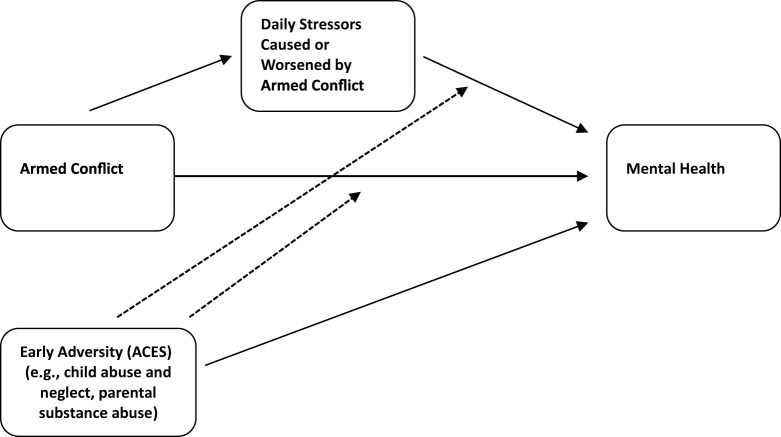
Ecological model of distress in conflict-affected populations with the addition of adverse childhood experiences (ACEs).

*Note*: Solid lines indicate causal pathways, dotted line indicates moderation

Ceccarelli and colleagues have observed that research on ACEs has been heavily concentrated in high-income countries; conversely, the impact of ACEs on mental health has been comparatively overlooked in research in low- and middle-income countries, where the great majority of conflict-affected populations live (Ceccarelli *et al.*, 2022). Even in research with refugees in high-income countries, however, we find the same inattention to early adversity as a possible influence on current mental health. The absence of ACEs in our model, and the neglect of ACEs in the literature on war and mental health, imply that war exposure and daily stressors are the critical variables impacting mental health in conflict-affected populations. Although this may be true, the predictive power of early adversity in populations unaffected by armed conflict makes it implausible that ACEs are not also relevant to understanding mental health in conflict-affected populations. We suspect the inattention to ACEs likely reflects a saliency bias, with attention drawn by the greater immediacy and visibility of war exposure and daily stressors relative to temporally distant experiences of childhood adversity. However, as we have discussed elsewhere (Rasmussen *et al.*, 2018), developmental trauma and heightened psychological vulnerability stemming from early adversity represent potentially important factors to be considered in research on armed conflict and mental health. The addition of ACEs to our model reflects this broadened conceptualization (see Fig. 2, adapted from Rasmussen *et al.*, 2018).

## Conclusion

The ecological model of distress in conflict-affected populations that we proposed fifteen years ago has found robust support in the ensuing years. It is by now well-established that the effects of armed conflict can only be understood by considering both direct exposure to the violence and destruction of war, and the stressful conditions of everyday life caused or exacerbated by organized violence. We feel cautiously optimistic in our appraisal that the model has contributed to bridging the once-contentious divide between advocates of what we have come to refer to as the war exposure and social-environmental frameworks. We recognize the pivotal role of numerous colleagues and other conceptual frameworks in helping to resolve this unhelpful and unnecessary conflict, and in gradually moving the field of MHPSS towards more integrated and multilevel programming, and more rigorous evaluations of social environmental interventions.

The model we sketched out in 2010 was intentionally minimalist and included just two sets of variables predicting distress: direct exposure to armed conflict, and daily stressors (including those caused or worsened by armed conflict, and those that exist independently of conflict). This simple model reflected our primary aim of integrating the two dominant frameworks at the time, which focused precisely on these two categories of stressors. Although the model offers some nuance by delineating the partial mediation of war exposure on mental health by daily stressors, it is nonetheless a simple model, perhaps overly so. As we have discussed above, it does not include moderators of the relationships between stressors and mental health outcomes. Nor does it acknowledge the bidirectional influence of distress generated by war exposure and daily stressors. Nonetheless, we are satisfied that the model has served its primary purpose of drawing attention to the essential complementarity of the war exposure and social-environmental approaches. We leave the elaboration of the model with regard to these and other possible gaps to others, with the exception of ACEs. Because research in high-income countries has consistently shown such a powerful effect of early adversity on future mental health, we have added ACEs to our model. We suggest that early adversity has both a direct effect on adult mental health in conflict-affected populations (as it does in other populations), and that it leaves people more vulnerable to the impact of exposure to potentially traumatic events (i.e., war exposure) as well as daily stressors; that is, we hypothesize that early adversity moderates the relationship of both war exposure and daily stressors to mental health, with greater adversity leading to heightened vulnerability. Whether this turns out to be correct is a question for future research to explore.
